# Supplementary material for: Neural encoding of semantic structures during sentence production
Source: Cereb Cortex. 2024 Dec 24;34(12):bhae482. doi: 10.1093/cercor/bhae482 (PMC11666472; doi:10.1093/cercor/bhae482)
Supplement: Supplementary_Information_bhae482 [file supplementary_information_bhae482.pdf]

Supplementary Information

fMRIprep preprocessing pipeline

Anatomical data preprocessing

The T1-weighted (T1w) image was corrected for intensity non-uniformity (INU) with N4BiasFieldCorrection, distributed with ANTs 2.3.3 (Avants et al. 2008) and used as T1w-reference throughout the workflow. The T1w-reference was then skull-stripped with a *Nipype* implementation of the antsBrainExtraction.sh workflow (from ANTs), using OASIS30ANTs as target template. Brain tissue segmentation of cerebrospinal fluid (CSF), white-matter (WM) and gray-matter (GM) was performed on the brain-extracted T1w using fast (FSL 5.0.9, Zhang et al., 2001). Brain surfaces were reconstructed using recon-all (FreeSurfer 6.0.1, Dale et al., 1999), and the brain mask estimated previously was refined with a custom variation of the method to reconcile ANTs-derived and FreeSurfer-derived segmentations of the cortical gray-matter of Mindboggle (Klein et al. 2017). Volume-based spatial normalization to MNI standard space (MNI152NLin2009cAsym) was performed through nonlinear registration with antsRegistration (ANTs 2.3.3), using brain-extracted versions of both T1w reference and the T1w template.

Functional data preprocessing

For each of the 6 BOLD runs per subject, the following preprocessing was performed. First, a reference volume and its skull-stripped version were generated using a custom methodology of *fMRIPrep*. A B0-nonuniformity map (or *fieldmap*) was estimated based on a phase-difference map. The *fieldmap* was then co-registered to the target EPI (echo-planar imaging) reference run and converted to a displacements field map. Based on the estimated susceptibility distortion, a corrected EPI (echo-planar imaging) reference was calculated for a more accurate co-registration with the anatomical reference. The BOLD reference was then co-registered to the T1w reference using bbregister (FreeSurfer) which implements boundary-based registration (Greve and Fischl 2009). Co-registration was configured with six degrees of freedom. Head-motion parameters with

respect to the BOLD reference (transformation matrices, and six corresponding rotation and translation parameters) are estimated before any spatiotemporal filtering using mcflirt (FSL 5.0.9, Jenkinson et al., 2002). The BOLD time-series were resampled into standard space, generating a *preprocessed BOLD run in MNI152NLin2009cAsym space*. First, a reference volume and its skull-stripped version were generated using a custom methodology of *fMRIPrep*. Automatic removal of motion artifacts using independent component analysis (ICA-AROMA, Pruim et al. 2015) was performed on the *preprocessed BOLD on MNI space* time-series after removal of non-steady state volumes and spatial smoothing with an isotropic, Gaussian kernel of 6mm FWHM (full-width half-maximum). The "aggressive" noise-regressors were collected for nuisance regression in first-level analysis. Several confounding time-series were calculated based on the *preprocessed BOLD*: framewise displacement (FD), the derivative of the relative (frame-to-frame) bulk head motion variance (DVARS) and three region-wise global signals. FD was computed using two formulations following Power (absolute sum of relative motions, Power et al. (2014)) and Jenkinson (relative root mean square displacement between affines, Jenkinson et al. (2002)). FD and DVARS are calculated for each functional run, both using their implementations in *Nipype* (following the definitions by Power et al. 2014). Additionally, a set of physiological regressors were extracted to allow for component-based noise correction (*CompCor*, Behzadi et al. 2007). Principal components are estimated after high-pass filtering the *preprocessed BOLD* time-series (using a discrete cosine filter with 128s cut-off). For anatomical CompCor, three probabilistic masks (CSF, WM and combined CSF+WM) are generated in anatomical space. The head-motion estimates calculated in the correction step were also placed within the corresponding confounds file. All resamplings can be performed with *a single interpolation step* by composing all the pertinent transformations (i.e. head-motion transform matrices, susceptibility distortion correction when available, and co-registrations to anatomical and output spaces). Gridded (volumetric) resamplings were performed using antsApplyTransforms (ANTs). Non-gridded (surface) resamplings were performed using mri_vol2surf (FreeSurfer).

Supplementary Table 1: *Statistics and peaks for whole-brain analysis for each encoding model during sentence production. Voxelwise p < 0.005, p < 0.05 FDR cluster corrected.*

Contrast	Cluster			Peak Voxel (MNI Coordinates)			Anatomical Location
	***p* (FWE-corrected)**	**Size**	**mean *z***	**x**	**y**	**z**	
**bag-of-nouns**	2.60E-05	6217	0.001391	-49	2	41	L Precentral Gyrus
	0.000286	1188	0.001447	2	12	51	Supplementary Motor Area
	0.011216	427	0.000847	26	-68	-51	R Cerebellum (VIIb)
	0.017631	330	0.000673	-22	10	-8	L Putamen
	0.017631	307	0.000793	68	-25	-6	R MTG
	0.035058	223	0.001185	61	-30	29	R Supramarginal Gyrus
**broad roles**	2.24E-05	1984	0.002009	-61	-45	24	L Supramarginal Gyrus
	4.48E-05	1259	0.002311	-51	2	41	L Precentral Gyrus
	0.002552	451	0.002305	-36	-60	56	L Superior Parietal Lobule
	0.002552	416	0.001809	56	-15	11	R Planum Temporale
	0.002552	414	0.002134	-2	12	54	Supplementary Motor Area
	0.021229	194	0.001937	1	32	36	Posterior Cingulate Cortex
	0.036216	147	0.002132	46	20	36	R Middle Frontal Gyrus
	0.036216	143	0.00152	6	45	-6	R Paracingulate Gyrus
	0.037371	135	0.00234	41	-72	36	R Superior Parietal Lobule
	0.038549	128	0.001733	-24	17	51	L Superior Frontal Sulcus
	0.03869	123	0.001432	46	-5	-1	R Opercular Cortex
	0.040269	117	0.001267	26	67	-51	R Cerebellum (VIIb)
**narrow roles**	0.000528	950	0.003602	-59	-17	6	L Planum Temporale
	0.005237	415	0.003266	3	27	56	Supplementary Motor Area
	0.018807	250	0.002346	61	-45	-11	R MTG
	0.025291	204	0.003363	56	-12	4	R Planum Temporale
	0.025291	179	0.003387	-2	25	39	L Paracingulate Gyrus
	0.025291	177	0.003133	26	10	2	R Putamen
	0.031269	155	0.003693	-31	42	36	L Anterior Middle Frontal Gyrus
	0.032171	146	0.003239	-14	-30	74	L dorsal Prefrontal Gyrus

Supplementary Table 2: *Statistics and peaks for whole-brain analysis for each encoding model during sentence comprehension. Voxelwise p < 0.005, p < 0.05 FDR cluster corrected.*

Contrast	Cluster			Peak Voxel (MNI Coordinates)			Anatomical Location
	p (FWE-corrected)	Size	mean *z*	x	y	z	
**bag-of-nouns**	0.000506	929	0.001026	-54	-40	26	L Supramarginal Gyrus
	0.007716	342	0.000964	-4	5	66	L Supplementary Motor Cortex
	0.007716	312	0.00105	-49	10	21	L Inferior Frontal Gyrus (opercularis)
	0.02089	200	0.000764	-46	29	-3	L Inferior Frontal Gyrus (triangularis)
	0.029061	158	0.00073	-49	-55	-13	L Inferior Temporal Gyrus
	0.029061	151	0.000788	53	15	21	R Inferior Frontal Gyrus
	0.040511	124	0.000977	-51	0	46	L Precentral Gyrus
**broad roles**	0.001772	753	0.001721	-49	32	16	L Inferior Frontal Gyrus (triangularis)
	0.002132	601	0.001559	-4	17	54	L Superior Frontal Gyrus
	0.003055	484	0.001522	-56	-65	11	L Middle Temporal Gyrus
	0.028618	204	0.000939	-46	-50	-16	L Inferior Temporal Gyrus
	0.028618	196	0.0018	43	-70	36	R Lateral Occipital Cortex
**narrow roles**	0.007365	436	0.003485	-49	32	14	L Inferior Frontal Gyrus (triangularis)
	0.019496	273	0.003841	-46	-70	36	L Angular Gyrus
	0.044796	175	0.003884	-59	-55	31	L Supramarginal Gyrus
	0.044796	163	0.003748	-11	-75	49	L Lateral Occipital Cortex
	0.044796	151	0.003402	51	25	29	R Middle Frontal Gyrus

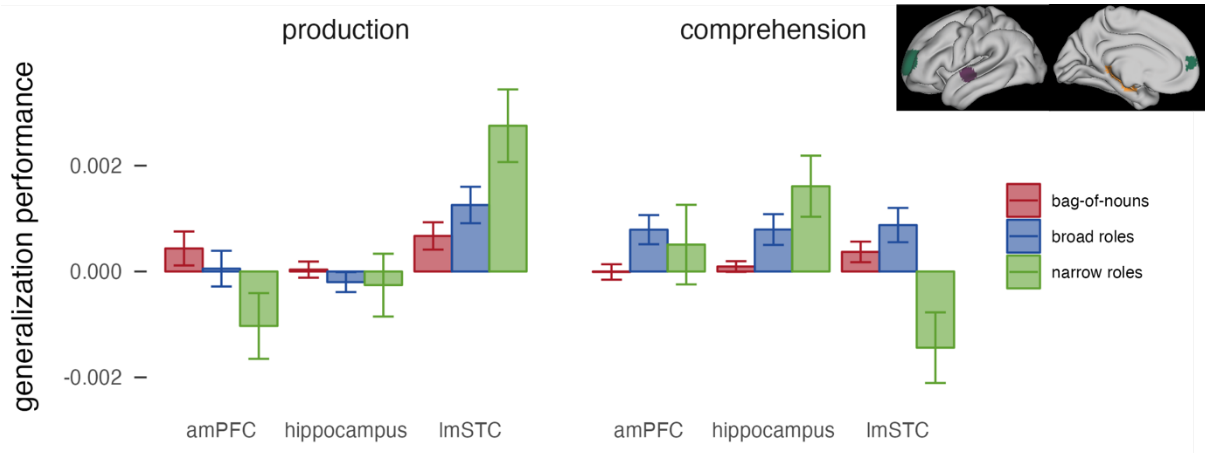

Supplementary Figure 1: Average generalization performance in three ROIs based on Frankland and Greene (2020a), selected independently for each participant, shown on the right. lmSTC: purple. amPFC: blue. Hippocampus: orange.
